# Statistical biases due to anonymization evaluated in an open clinical dataset from COVID-19 patients

**DOI:** 10.1038/s41597-022-01669-9

**Published:** 2022-12-21

**Authors:** Carolin E. M. Koll, Sina M. Pütz, Thierry Meurers, Chin Huang Lee, Mirjam Kohls, Christoph Stellbrink, Charlotte Thibeault, Lennart Reinke, Sarah Steinbrecher, Stefan Schreiber, Lazar Mitrov, Sandra Frank, Olga Miljukov, Johanna Erber, Johannes C. Hellmuth, Jens-Peter Reese, Fridolin Steinbeis, Thomas Bahmer, Marina Hagen, Patrick Meybohm, Stefan Hansch, István Vadász, Lilian Krist, Steffi Jiru-Hillmann, Fabian Prasser, Jörg Janne Vehreschild, NAPKON Study Group, I. Bernemann, T. Illig, M. Kersting, N. Klopp, V. Kopfnagel, S. Muecke, G. Anton, M. Kraus, A. Kuehn-Steven, S. Kunze, M. K. Tauchert, J. Vehreschild, M. Brechtel, S. Fuhrmann, S. M. Pütz, C. E. M. Koll, C. Lee, L. Mitrov, S. M. Nunes de Miranda, M. Nunnendorf, G. Sauer, K. Seibel, M. Stecher, K. Appel, R. Geisler, M. Hagen, M. Scherer, J. Schneider, C. Weismantel, B. Balzuweit, S. Berger, M. Hummel, S. Schmidt, M. Witzenrath, T. Zoller, A. Krannich, F. Kurth, J. Lienau, R. Lorbeer, C. Pley, J. Schaller, C. Thibeault, C. Bauer, C. Fiessler, M. Goester, A. Grau, P. Heuschmann, A. L. Hofmann, S. Jiru-Hillmann, K. Kammerer, M. Kohls, O. Miljukov, J. P. Reese, K. Ungethuem, M. Krawczak, J. C. Hellmuth, T. Bahls, W. Hoffmann, M. Nauck, C. Schäfer, M. Schattschneider, D. Stahl, H. Valtentin, I. Chaplinskaya, S. Hanß, D. Krefting, C. Pape, J. Hoffmann, M. Hummel, S. Schmidt, M. Witzenrath, T. Zoller, J. C. Hellmuth, J. Fricke, T. Helbig, T. Keil, L. Kretzler, L. Krist, L. Lippert, M. Mittermaier, M. Mueller-Plathe, M. Roennefarth, L. E. Sander, F. Steinbeis, S. Steinbrecher, D. Treue, P. Triller, S. Zvorc, F. Hammer, L. Horvarth, A. Kipet, M. Schroth, M. T. Unterweger, I. Bernemann, N. Drick, M. Hoeper, T. Illig, M. Kersting, N. Klopp, V. Kopfnagel, I. Pink, M. Ratowski, F. Zetzsche, C. M. Bremer, H. H. Halfar, S. Herold, L. H. Nguyen, C. Ruppert, M. Scheunemann, W. Seeger, A. Uribe Munoz, I. Vadasz, M. Wessendorf, H. Azzaui, M. Gräske, M. Hower, J. Kremling, E. Landsiedel-Mechenbier, A. Riepe, B. Schaaf, S. Frank, M. Huber, S. Kaeaeb, O. T. Keppler, E. Khatamzas, C. Mandel, S. Mueller, M. Muenchhoff, L. Reeh, C. Scherer, H. Stubbe, M. von Bergwelt, L. Weiß, B. Zwißler, M. Milovanovic, R. Pauli, M. Ebert, W. K. Hofmann, M. Neumaier, F. Siegel, A. Teulfel, C. Wyen, C. Allerlei, A. Keller, J. Walter, R. Bals, C. Herr, M. Krawczyk, C. Lensch, P. M. Lepper, M. Riemenschneider, S. Smola, M. Zemlin, C. Raichle, G. Slesak, S. Bader, J. Classen, C. Dhillon, M. Freitag, V. Gruenherz, B. Maerkl, H. Messmann, C. Roemmele, M. Steinbrecher, M. Ullrich, H. Altmann, R. Berner, S. Dreßen, T. Koch, D. Lindemann, K. Seele, P. Spieth, K. Tausche, N. Toepfner, S. von Bonin, D. Kraska, A. E. Kremer, M. Leppkes, J. Mang, M. F. Neurath, H. U. Prokosch, J. Schmid, M. Vetter, C. Willam, K. Wolf, M. Addo, A. L. F. Engels, D. Jarczak, M. Kerinn, S. Kluge, R. Kobbe, K. Roedl, C. Schlesner, P. Shamsrizi, T. Zeller, C. Arendt, C. Bellinghausen, S. Cremer, A. Groh, A. Gruenewaldt, Y. Khodamoradi, S. Klinsing, G. Rohde, M. Vehreschild, T. Vogl, K. Becker, M. Doerr, K. Lehnert, M. Nauck, N. Piasta, C. Schaefer, E. Schaefer, M. Schattschneider, C. Scheer, D. Stahl, R. Baber, S. Bercker, N. Krug, S. D. Mueller, H. Wirtz, G. Boeckel, J. A. Meier, T. Nowacki, P. R. Tepasse, R. Vollenberg, C. Wilms, A. Arlt, F. Griesinger, U. Guenther, A. Hamprecht, K. Juergens, A. Kluge, C. Meinhardt, K. Meinhardt, A. Petersmann, R. Prenzel, A. Brauer-Hof, C. Brochhausen-Delius, R. Burkhardt, M. Feustel, F. Hanses, M. Malfertheiner, T. Niedermair, B. Schmidt, P. Schuster, S. Wallner, D. Mueller-Wieland, N. Marx, M. Dreher, E. Dahl, J. Wipperfuerth, T. Bahmer, J. Enderle, A. Friedrichs, A. Hermes, N. Kaeding, M. Koerner, M. Krawczak, C. Kujat, I. Lehmann, M. Lessing, W. Lieb, C. Maetzler, M. Oberländer, D. Pape, M. Plagge, L. Reinke, J. Rupp, S. Schreiber, D. Schunk, L. Tittman, W. Barkey, J. Erber, L. Fricke, J. Lieb, T. Michler, L. Mueller, J. Schneider, C. Spinner, F. Voit, C. Winter, M. Bitzer, S. Bunk, S. Göpel, H. Häberle, K. Kienzle, H. Mahrhofer, N. Malek, P. Rosenberger, C. Struemper, F. Trauner, S. Frantz, A. Frey, K. Haas, C. Haertel, K. G. Haeusler, G. Hein, J. Herrmann, A. Horn, N. Isberner, R. Jahns, M. Kohls, J. Liese, P. Meybohm, C. Morbach, J. Schmidt, P. Schulze, S. Stoerk, B. Weissbrich, F. Brinkmann, Y. Brueggemann, T. Gambichler, K. Hellwig, T. Luecke, A. Reinacher-Schick, W. E. Schmidt, C. Schuette, E. Steinmann, C. Torres Reyes, K. Alsaad, B. Berger, E. Hamelmann, H. Heidenreich, C. Hornberg, N. S. A. Kulamadayil-Heidenreich, P. Maasjosthusmann, A. Muna, C. Olariu, B. Ruprecht, J. Schmidt, C. Stellbrink, J. Tebbe, D. August, M. Barrera, V. Goetz, A. Imhof, S. Koch, A. Nieters, G. Peyerl-Hoffmann, S. R. Rieg, A. Amanzada, S. Blaschke, A. Hafke, G. Hermanns, M. Kettwig, O. Moerer, S. Nussbeck, J. Papenbrock, M. Santibanez-Santana, S. Zeh, S. Dolff, C. Elsner, A. Krawczyk, R. J. Madel, M. Otte, L. Brochhagen, O. Witzke, J. Vehreschild, M. Witzenrath, P. Heuschmann, W. Hoffmann, T. Illig, S. Herold, R. Heyder, H. Neuhauser, S. Schreiber, M. von Lilienfeld-Toal, J. Vehreschild, M. Witzenrath, P. Heuschmann, T. Illig, S. Frank, A. Petersmann, P. Meybohm, S. Blaschke, S. Schreiber, M. von Lilienfeld-Toal, C. Ellert, A. Friedrichs, K. Milger, G. Schmidt, O. Witzke

**Affiliations:** 1 University of Cologne, Faculty of Medicine and University Hospital Cologne, Department I of Internal Medicine, Center for Integrated Oncology Aachen Bonn Cologne Duesseldorf, Cologne, Germany; 2 Berlin Institute of Health at Charité – Universitätsmedizin Berlin, Charitéplatz 1, 10117 Berlin, Germany; 3 University of Wuerzburg, Faculty of Medicine, Institute for Clinical Epidemiology and Biometry, Wuerzburg, Germany; 4 Department of Cardiology and Intensive Care Medicine, Bielefeld Medical Centre, Medical Faculty OWL, University of Bielefeld, Bielefeld, Germany; 5 Charité – Universitätsmedizin Berlin, corporate member of Freie Universität Berlin and Humboldt Universität zu Berlin, Berlin, Germany; 6 Internal Medicine Department I, University Medical Center Schleswig-Holstein Campus Kiel, Kiel, Germany; 7 Department of Anesthesiology, University Hospital of Ludwig-Maximilians-University (LMU), Munich, Germany; 8 Department of Medicine III, University Hospital, LMU Munich, Munich, Germany; 9 Technical University of Munich, School of Medicine, University Hospital rechts der Isar, Department of Internal Medicine II, Munich, Germany; 10 COVID-19 Registry of the LMU Munich (CORKUM), University Hospital, LMU Munich, Munich, Germany; 11 Airway Research Center North (ARCN), German Center for Lung Research (DZL), Großhansdorf, Germany; 12 Department II for Internal Medicine, Hematology/Oncology, University Hospital Frankfurt, Frankfurt am Main, Germany; 13 Department of Anaesthesiology, Intensive Care, Emergency and Pain Medicine, University Hospital Wuerzburg, Wuerzburg, Germany; 14 Department of Infection Prevention and Infectious Diseases, University Hospital Regensburg, Regensburg, Germany; 15 Department of Internal Medicine, Justus Liebig University, Universities of Giessen and Marburg Lung Center (UGMLC), Member of the German Center for Lung Research (DZL), Giessen, Germany; 16 The Cardio-Pulmonary Institute (CPI), Giessen, Germany; 17 Institute of Social Medicine, Epidemiology and Health Economics, Charité-Universitätsmedizin Berlin, Berlin, Germany; 18 German Centre for Infection Research (DZIF), partner site Bonn-Cologne, Cologne, Germany; 19 Hannover Unified Biobank, Hannover Medical School, Hannover, Germany; 20 Institute of Epidemiology, Helmholtz Center Munich, Munich, Germany; 21 University Hospital Cologne and University Hospital Frankfurt, Cologne and Frankfurt, Germany; 22 University Hospital Cologne, Cologne, Germany; 23 University Hospital Frankfurt, Frankfurt am Main, Germany; 24 Charité - Universitaetsmedizin Berlin, Berlin, Germany; 25 University of Wuerzburg, Wuerzburg, Germany; 26 University of Kiel, Kiel, Germany; 27 University Hospital LMU Munich, Munich, Germany; 28 University Medicine Greifswald, Greifswald, Germany; 29 University Medical Center Goettingen, Goettingen, Germany; 30 German Center for Cardiovascular Diseases (DZHK), Berlin, Germany; 31 Cnopfsche Children’s Hospital, Nuernberg, Germany; 32 Hannover Medical School, Hannover, Germany; 33 Justus Liebig University Giessen & Marburg, Gießen and Marburg, Germany; 34 Klinikum Dortmund, Dortmund, Germany; 35 Malteser Hospital St. Franziskus Hospital, Flensburg, Germany; 36 MVZ am Isartor, Muenchen, Germany; 37 Medical Faculty Mannheim, Mannheim, Germany; 38 Practice for general medicine Am Ebertplatz, Cologne, Germany; 39 Practice for general medicine Dr. Allerlei, Frankfurt am Main, Germany; 40 Saarland University, Homburg, Germany; 41 Saarland University Hospital, Homburg, Germany; 42 Tropical Hospital Paul-Lechler-Krankenhaus, Tuebingen, Germany; 43 University Hospital Augsburg, Augsburg, Germany; 44 University Hospital Carl Gustav Carus, Dresden, Germany; 45 University Hospital Erlangen, Erlangen, Germany; 46 University Hospital Hamburg-Eppendorf, Hamburg, Germany; 47 University Hospital Greifswald, Greifswald, Germany; 48 University Hospital Leipzig, Leipzig, Germany; 49 University Hospital Muenster, Muenster, Germany; 50 University Medicine Oldenburg, Oldenburg, Germany; 51 University Hospital Regensburg, Regensburg, Germany; 52 University Hospital RWTH Aachen, Aachen, Germany; 53 University Hospital Schleswig-Holstein, Kiel and Luebeck, Germany; 54 University Hospital Technical University Munich, Munich, Germany; 55 University Hospital Tuebingen, Tuebingen, Germany; 56 University Hospital Wuerzburg, Wuerzburg, Germany; 57 University Hospitals of the Ruhr University Bochum, Bochum, Germany; 58 Bielefeld University, Medical School and University Medical Center East Westphalia-Lippe, Bielefeld, Germany; 59 University Medical Center Freiburg, Freiburg, Germany; 60 University Medicine Essen, Essen, Germany; 61 University Hospital Giessen and Marburg, Giessen, Germany; 62 Robert Koch Institute, Department of Epidemiology and Health Monitoring, Berlin, Germany; 63 University Hospital Schleswig-Holstein, Kiel, Germany; 64 Jena University Hospital, Jena, Germany; 65 Lahn-Dill-Clinics, Wetzlar, Germany; 66 University Hospital Essen, Essen, Germany

**Keywords:** Medical research, Diseases

## Abstract

Anonymization has the potential to foster the sharing of medical data. State-of-the-art methods use mathematical models to modify data to reduce privacy risks. However, the degree of protection must be balanced against the impact on statistical properties. We studied an extreme case of this trade-off: the statistical validity of an open medical dataset based on the German National Pandemic Cohort Network (NAPKON), which was prepared for publication using a strong anonymization procedure. Descriptive statistics and results of regression analyses were compared before and after anonymization of multiple variants of the original dataset. Despite significant differences in value distributions, the statistical bias was found to be small in all cases. In the regression analyses, the median absolute deviations of the estimated adjusted odds ratios for different sample sizes ranged from 0.01 [minimum = 0, maximum = 0.58] to 0.52 [minimum = 0.25, maximum = 0.91]. Disproportionate impact on the statistical properties of data is a common argument against the use of anonymization. Our analysis demonstrates that anonymization can actually preserve validity of statistical results in relatively low-dimensional data.

## Introduction

The Severe Acute Respiratory Syndrome Coronavirus II (SARS-CoV-2) pandemic has now been ongoing for more than two years^1–3^. On 15th of March 2022, worldwide, more than 460 million infected cases have been detected and more than 6 million people have died as a result of the Coronavirus Disease 2019 (COVID-19, https://covid19.who.int/). In order to collect high-quality clinical data, image data and biosamples of COVID-19 patients in Germany, the National Pandemic Cohort Network (NAPKON) was founded in 2020 as part of the Network University Medicine (NUM), a state-funded network to tackle the COVID-19 pandemic in Germany^4^. NAPKON consists of three sub-cohorts, which differ in granularity, severity of disease and sector of recruitment (Cross-Sectoral Platform (SUEP), High-Resolution Platform (HAP), and Population-Based Platform (POP)). In NAPKON, extensive clinical data have been collected during the acute course of COVID-19 and the post-COVID-19 phase, reaching in total over 4000 variables in the SUEP and HAP and over 2000 variables in the POP.

To make important parameters on the clinical course and outcome of COVID-19 openly available without restrictions, a public clinical dataset (Public Use File, PUF) with patient-level information was developed within NAPKON. The dataset is updated on a monthly basis and can be downloaded on the project website (https://napkon.de/statistik/). Most public COVID-19 datasets were generated from governmental sources or contain aggregated data only^5–9^. The NAPKON PUF combines quality-controlled clinical parameters from cohort studies or clinical routine, such as severity of disease, with demographic information, such as age and gender. Access to comprehensive data and biosamples from NAPKON can be requested by internal and external scientists through clearly defined use and access procedure. Although requests are processed promptly, they do not allow immediate access, which has been described as an effective strategy to fight the COVID-19 pandemic^10^. In addition, the reason for access and goal of data usage must be defined in advance. The NAPKON PUF provides an openly accessible overview of the NAPKON cohorts in near real-time. This simplifies the preparations for more complex data analyses and makes the cohort data more accessible for international scientists.

In NAPKON, to ensure that publishing the PUF does not compromise the privacy of individuals, the data is processed through an anonymization pipeline. The pipeline uses mathematical and statistical privacy models preventing re-identification of individuals, singling out as well as the inference of sensitive information. Records of individuals whose publication would not meet the privacy guarantees specified are withhold from the dataset. The anonymization pipeline is based on the approach used in the LEOSS project that has already been successfully used for releasing data about over 10,000 patients to the public^11^.

Although data anonymization can significantly contribute to protecting the privacy of individuals, modifying the data can lead to a reduction of its usefulness. While the anonymization process implemented for NAPKON contains optimization procedures to minimize the loss of information, the extent to which the transformations performed impact the usefulness of the dataset can only be investigated in the context of specific usage scenarios. The objective of this work was to evaluate whether and how the anonymization process used in the creation of the NAPKON PUF affects its statistical properties with regard to the three NAPKON sub-cohorts. To address this question, we performed multiple evaluations on the dataset before and after anonymization. The evaluations included descriptive statistics and regression models. Thereafter, we assessed the extent of bias introduced by the anonymization process used in our results and thus its effects on the dataset’s usefulness.

## Results

### Anonymized clinical dataset

The NAPKON PUF used in this study contained clinical data from 3,904 cases captured in 15 variables. Following a qualitative analysis of the attributes contained in the dataset, the risk of linkage or singling out was controlled by reducing the uniqueness of combinations of the variables age, gender, quarter and year of diagnosis, and cohort. The risk of sensitive attribute inference was further controlled for the variables defining the severity of disease, the patient status at the end of acute phase, presence of intensive care treatment or invasive ventilation, and ability and any symptoms at three months follow up. Details can be found in the methods section.

### Fraction of cases published

**Fig. 1:**
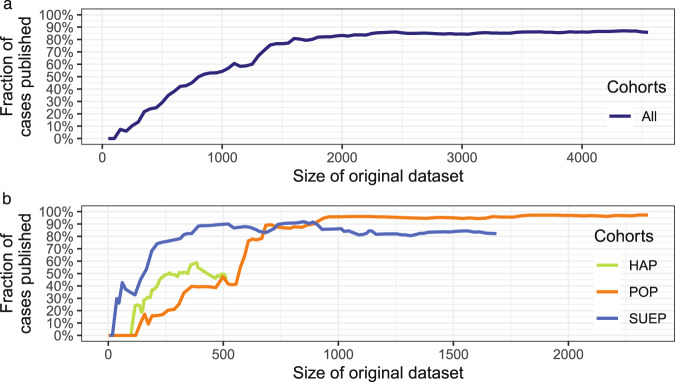
Fraction of cases published for the complete NAPKON dataset **(a)** and **(b)** the High-Resolution Platform (HAP), the Population-based Platform (POP), and the Cross-Sectoral Platform (SUEP).

The PUF contained a subset of the cases present in the original dataset, as the anonymization process was configured to withhold cases from release for which the defined privacy guarantees would not hold. Figure 1 provides an overview of the number of cases included in the PUF for increasing sizes of the dataset and for the three sub-cohorts within NAPKON. The size of the original dataset has increased with the time the registry was running.

The fraction of cases from the NAPKON cohort that can be included in the PUF increased to over 85% as soon as at least 2,250 cases were documented (which happened on 2021-05-18). The curve flattened out at 1,600 documented cases. The highest fraction of cases included in the PUF (87%) was reached when 4,350 cases were documented. The absolute number of cases in the PUF was reduced due to anonymization from 1,697 to 1,410 (83%) for the SUEP, from 2,346 to 2,280 (97%) for the POP, and from 519 to 237 (45%) for the HAP.

### Descriptive statistics before and after anonymization

**Fig. 2:**
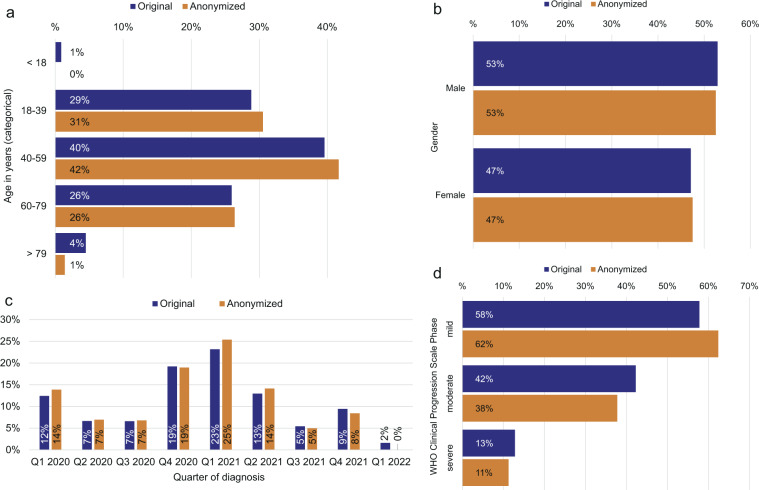
Comparison of demographic parameters of patients for the original dataset (n = 4,562) and the anonymized dataset (PUF; n = 3,904). The proportions are given in percentage. Note: The percentages in the PUF may be larger if the number of censored cases is unbalanced. (**a**) Age distribution in years, (**b**) gender distribution, (**c**) distribution of quarter and year of first positive SARS-CoV-2 test, and (**d**) distribution of the disease severity in the course of disease. WHO = World Health Organization.

**Fig. 3:**
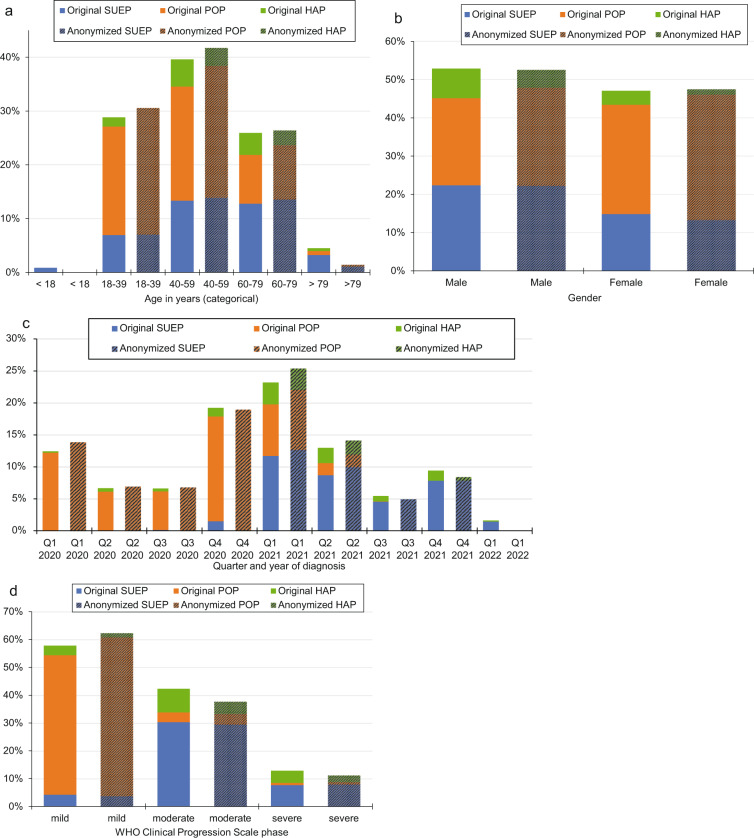
Comparison of demographic parameters of patients for the original data set (n = 4,562) and the anonymized data set (PUF, n = 3,904). (**a**) Age distribution in years, (**b**) gender distribution, (**c**) distribution of quarter and year of first positive SARS-CoV-2 test, and (**d**) distribution of the disease severity in the course of disease. HAP = High-Resolution Platform; POP = Population-based Platform; SUEP = Cross-Sectoral Platform; WHO = World Health Organization.

Table 1 shows the cohort descriptions from the original dataset from 2022-03-15 (n = 4,562) and the PUF (n = 3,904) for each of the three NAPKON cohorts. The age distribution (Fig. 2(a)) before and after anonymization differed significantly (P < 0.001). It is notable that in particular the age groups under 18 and over 79 years were represented with only a few cases in the original dataset, which is why no minors were included in the PUF and the age group over 79 years was reduced by 73% in size (150/205) through the anonymization process. In contrast, the sizes of the other age groups differed only slightly between the two datasets. The largest impact can be observed for the HAP cohort (Fig. 3). Only 56% (129/230) of its cases in the age group 40–59 and 58% (108/187) of its cases in the age group 60–79, were included in the PUF. The POP shows the smallest case reduction regarding the variable age, however with a significantly different age distribution (P = 0.011). The age category over 79 years stands out with a case reduction of 66% (12/35).

**Table 1:** Description of the three NAPKON sub-cohorts in the original and anonymized (PUF) dataset from 2022-03-15.

	Original (n = 4,562)			Anonymized (n = 3,904)		
	SUEP (n = 1,697)	POP (n = 2,346)	HAP (n = 519)	SUEP (n = 1,387)	POP (n = 2,280)	HAP (n = 237)
**Age in years**						
<18	40 (2.4%)	0 (0%)	0 (0%)	0 (0%)	0 (0%)	0 (0%)
18–39	317 (18.7%)	920 (39.2%)	78 (15.0%)	275 (19.8%)	917 (40.2%)	0 (0%)
40–59	609 (35.9%)	967 (41.2%)	230 (44.3%)	541 (39.0%)	957 (42.0%)	129 (54.4%)
60–79	584 (34.4%)	412 (17.6%)	187 (36.0%)	528 (38.1%)	394 (17.3%)	108 (45.6%)
>79	147 (8.7%)	35 (1.5%)	23 (4.4%)	43 (3.1%)	12 (0.5%)	0 (0%)
Missing	0 (0%)	12 (0.5%)	1 (0.2%)	0 (0%)	0 (0%)	0 (0%)
**Gender**						
Male	1,020 (60.1%)	1,040 (44.3%)	352 (67.8%)	868 (62.6%)	1001 (43.9%)	181 (76.4%)
Female	677 (39.9%)	1,305 (55.6%)	166 (32.0%)	519 (37.4%)	1,279 (56.1%)	56 (23.6%)
Missing	0 (0%)	1 (0.04%)	1 (0.2%)	0 (0%)	0 (0%)	0 (0%)
**Quarter and year of first COVID-19 diagnosis**						
Q1 2020	2 (0.1%)	554 (23.6%)	11 (2.1%)	0 (0%)	541 (23.7%)	0 (0%)
Q2 2020	0 (0%)	279 (11.9%)	24 (4.6%)	0 (0%)	271 (11.9%)	0 (0%)
Q3 2020	7 (0.4%)	275 (11.7%)	19 (3.7%)	0 (0%)	265 (11.6%)	0 (0%)
Q4 2020	68 (4.0%)	748 (31.9%)	61 (11.8%)	0 (0%)	740 (32.4%)	0 (0%)
Q1 2021	534 (31.5%)	367 (15.6%)	156 (30.1%)	496 (35.8%)	365 (16.0%)	130 (54.9%)
Q2 2021	397 (23.4%)	86 (3.7%)	108 (20.8%)	390 (28.1%)	76 (3.3%)	85 (35.9%)
Q3 2021	208 (12.3%)	1 (0.04%)	39 (7.5%)	194 (14.0%)	0 (0%)	0 (0%)
Q4 2021	357 (21.0%)	1 (0.04%)	72 (13.9%)	307 (22.1%)	0 (0%)	22 (9.3%)
Q1 2022	66 (3.9%)	0 (0%)	7 (1.3%)	0 (0%)	0 (0%)	0 (0%)
Missing	58 (3.4%)	35 (1.5%)	22 (4.2%)	0 (0%)	22 (1.0%)	0 (0%)
**WHO Clinical Progression Scale most severe phase**						
Mild	191 (11.3%)	2185 (93.1%)	0 (0%)	149 (10.7%)	2131 (93.5%)	0 (0%)
Moderate	1,135 (66.9%)	124 (5.3%)	320 (61.7%)	919 (66.3%)	114 (5.0%)	144 (60.8%)
Severe	349 (20.6%)	37 (1.6%)	199 (38.3%)	307 (22.1%)	35 (1.5%)	93 (39.2%)
Missing	22 (1.3%)	0 (0%)	0 (0%)	12 (0.9%)	0 (0%)	0 (0%)
**Patient status at end acute phase**						
Ambulant	192 (11.3%)	2,108 (89.9%)	0 (0%)	149 (10.7%)	2,069 (90.7%)	0 (0%)
Discharged	1,084 (63.9%)	124 (5.3%)	407 (78.4%)	911 (65.7%)	112 (4.9%)	180 (75.9%)
Referral/transfer	221 (13.0%)	0 (0%)	26 (5.0%)	176 (12.7%)	0 (0%)	14 (5.9%)
Dead	103 (6.1%)	0 (0%)	62 (11.9%)	87 (6.3%)	0 (0%)	34 (14.3%)
Missing	97 (5.7%)	114 (4.9%)	23 (4.4%)	64 (4.6%)	99 (4.3%)	9 (3.8%)
**Hospitalization**						
Yes	1,502 (88.5%)	161 (6.9%)	519 (100%)	1,238 (89.3%)	149 (6.5%)	237 (100%)
No	192 (11.3%)	2,185 (93.1%)	0 (0%)	149 (10.7%)	2,131 (93.5%)	0 (0%)
Missing	3 (0.2%)	0 (0%)	0 (0%)	0 (0%)	0 (0%)	0 (0%)
**Intensive Care Treatment**						
Yes	403 (23.7%)	33 (1.4%)	200 (38.5%)	356 (25.7%)	31 (1.4%)	99 (41.8%)
No	1,294 (76.3%)	2,313 (98.6%)	319 (61.5%)	1,031 (74.3%)	2,249 (98.6%)	138 (58.2%)
**Invasive ventilation**						
Yes	153 (9.0%)	18 (0.8%)	111 (21.4%)	128 (9.2%)	18 (0.8%)	60 (25.3%)
No	1,522 (89.7%)	2,328 (99.2%)	327 (63.0%)	1,245 (89.8%)	2,262 (99.2%)	131 (55.3%)
Missing	22 (1.3%)	0 (0%)	81 (15.6%)	14 (1.0%)	0 (0%)	46 (19.4%)
**3-month follow-up available**						
Yes	968 (57.0%)	2,346 (100%)	158 (30.4%)	859 (63.0%)	2,280 (100%)	103 (43.5%)
No/not yet	729 (43.0%)	0 (0%)	361 (69.6%)	528 (38.1%)	0 (0%)	134 (56.5%)
**Any symptoms at 3-month follow-up (if 3-month follow-up available)**						
Yes	305 (31.5%)	900 (38.4%)	43 (27.2%)	280 (32.6%)	874 (38.3%)	36 (35.0%)
No	663 (68.5%)	862 (36.7%)	115 (72.8%)	579 (67.4%)	847 (37.1%)	67 (65.0%)
Missing	0 (0%)	584 (24.9%)	0 (0%)	0 (0%)	559 (24.5%)	0 (0%)
**Ability to work at 3-month follow-up (if 3-month follow-up available)**						
Yes	454 (46.9%)	1,754 (74.8%)	41 (26.0%)	442 (51.5%)	1,729 (75.8%)	30 (29.1%)
No	89 (9.2%)	126 (5.4%)	91 (57.6%)	85 (10.0%)	124 (5.4%)	59 (57.3%)
Missing	425 (43.9%)	466 (19.9%)	26 (16.5%)	332 (38.7%)	427 (18.7%)	14 (13.6%)

3-month follow-up obtained 10 to 14 weeks after day of first COVID-19 diagnosis (for POP retrospective documentation). SUEP = Cross-Sectoral Platform; POP = Population-Based Platform; HAP = High-Resolution Platform.

The gender distribution is shown in Fig. 2(b). Males were predominant in the original (2,412/4,562; 53%) and anonymized (2,050/3,904, 53%) dataset. The gender distribution did not differ significantly before and after anonymization (P = 0.74). Analyzing the three cohorts (Fig. 3), 85% (868/1,020) of male cases could be published for the SUEP, 96% (1,001/1,040) for the POP and 51% (181/352) for the HAP. Consequently, gender distribution differed significantly in the HAP (P = 0.023), but not in the SUEP (P = 0.172) and in the POP (P = 0.753). The cases with a first COVID-19 diagnosis in 2020 were mainly from the POP and in 2021 from the SUEP (Fig. 2(c)). No significantly different distribution of quarter of diagnosis before and after anonymization could be observed for 2020 (P = 0.477). For 2021, the distribution differed significantly (P = 0.044) with regard to all cohorts, but not for cases in the SUEP (P = 0.66). In the first quarter of 2022 the case number was still too low so that no cases were included in the anonymized dataset.

The distribution of the documented disease phases was significantly different between the original and the anonymized dataset, with mild phases overrepresented and moderate phases underrepresented (P < 0.001, Fig. 2(d)). The proportion of included cases in the PUF differed for the POP, HAP, and SUEP. 97% (2,228/2,286) of cases from the POP having a documented mild COVID-19 phase (ambulatory treatment) were included in the PUF. A percentage of 84% (1,154/1,382) of cases with a documented moderate phase and 89% (312/349) with a documented severe phase were included in the dataset after anonymization from the SUEP and 44% (171/385) and 47% (93/199), respectively, from the HAP (Fig. 3(d)).

**Fig. 4:**
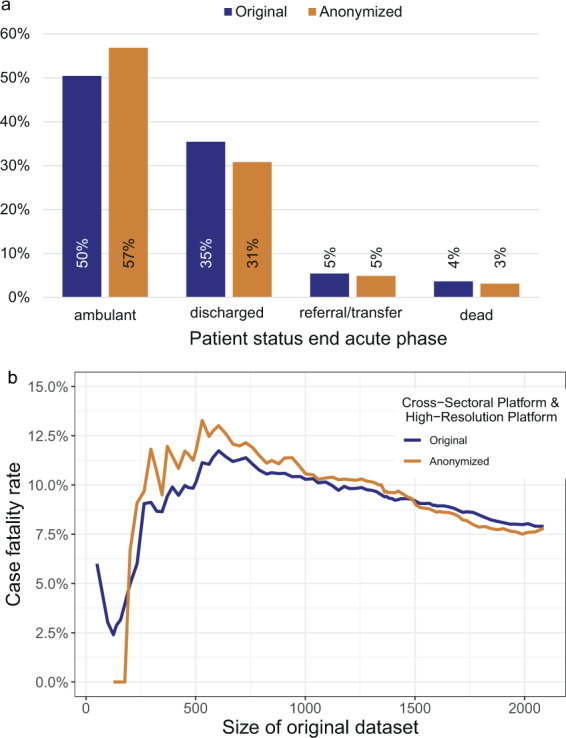
Comparison of patient status at end of acute phase before and after anonymization (anonymized dataset = PUF). The proportions are given in percentage. Note: The percentages in the PUF may be larger if the number of censored cases is unbalanced. (**a**) Distribution for the original dataset containing n = 4,562 and resulting anonymized dataset (n = 3,904). (**b**) Case fatality rates (patient status dead) are computed for the Cross-Sectoral Platform (SUEP) and High- Resolution Platform (HAP) cohorts over different sizes of original dataset. In the plot, the size of the original dataset is adjusted by the number of HAP and SUEP patients. To note, the Population-based Platform (POP) has recruited patients that survived SARS-CoV-2 infection only.

**Fig. 5:**
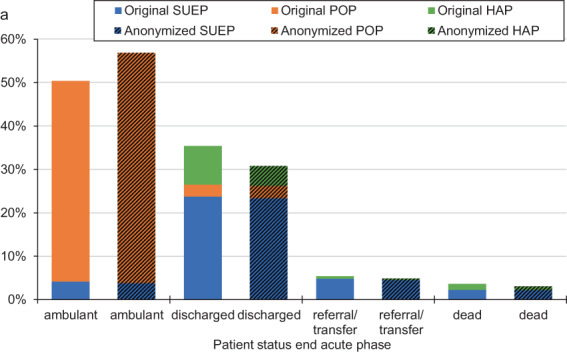
Comparison of patient status at end of acute phase before and after anonymization. Distribution for the original dataset containing n = 4,562 and resulting anonymized dataset (PUF, n = 3,904). HAP = High-Resolution Platform; POP = Population-based Platform; SUEP = Cross-Sectoral Platform.

The distribution of patient status at the end of the acute phase significantly differed with more cases in an ambulatory setting and less cases discharged, transferred, or died in the anonymized dataset in comparison to the original dataset (P < 0.001, Fig. 4(a)). Especially for the HAP, only 44% (180/407) of the cases with status “discharged” in the original dataset were added to the anonymized dataset (Fig. 5). For the POP, most cases were outpatients and only 2% of cases were removed from the original dataset.

### Case fatality rate

**Fig. 6:**
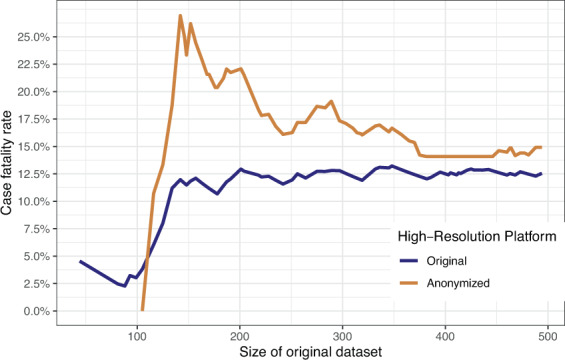
Case fatality rate for the High-Resolution Platform (HAP). Anonymized dataset = PUF.

**Fig. 7:**
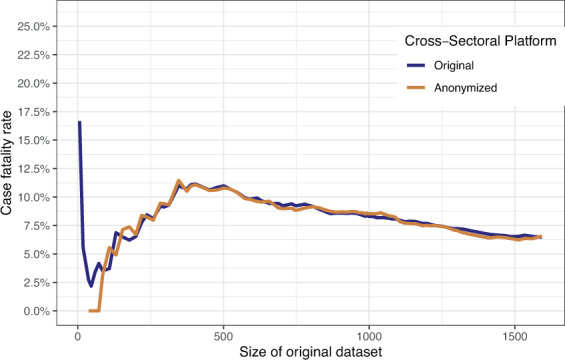
Case fatality rate for the Cross-Sectoral Platform (SUEP). Anonymized dataset = PUF.

We computed the case fatality rate for the SUEP and HAP cohorts (no fatalities in POP at first visit) for different sizes of the NAPKON dataset, showing the impact of the anonymization procedure on increasing documented cases over time (Fig. 4(b)). The case fatality rate was overestimated in the anonymized dataset with a size of up to 1,486 cases. For more than 1,486 cases documented in NAPKON, the case fatality rate was slightly underestimated in the anonymized dataset. In the original dataset containing 2,096 cases (dataset with three cohorts n = 4,562, 120 with missing information) the case fatality rate was 8% before and after anonymization (before: 165/2,096, after: 121/1,551). With more cases included in the original dataset, the bias of the case fatality rate before and after anonymization decreased. The differences in bias observed for the different cohorts is presented in Figs. 6 and 7. The median difference of the case fatality rate before and after anonymization for different sizes of the NAPKON datasets was 0.2% for the SUEP (interquartile range [IQR]: 0.1%–0.3%) and 2.9% (IQR: 1.7%–5.1%) for the HAP.

### Regression analyses before and after anonymization

**Fig. 8:**
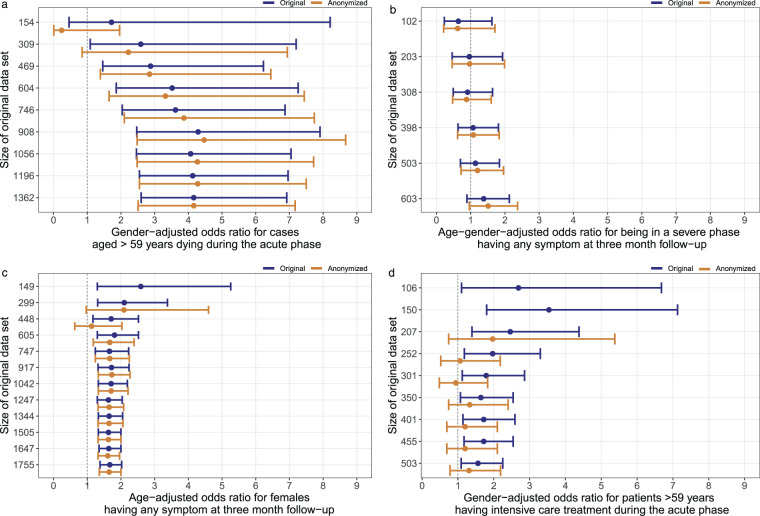
Odds ratios (OR) and 95%-confidence intervals (CI) of patient characteristics and outcomes in the dataset before and after anonymization for different sizes of the original dataset (anonymized dataset = PUF). In the graphs, the number of records in the original dataset was adjusted according to the number of cases included in the regression analysis, excluding missing data. To note, datasets with no ORs and CIs do not contain the relevant information for the respective regression model. **(a)** Inpatient cases from the Cross-Sectoral Platform (SUEP). **(b)** Cases from the High-Resolution Platform (HAP) and inpatient SUEP aged between 49 and 59 years that survived the acute phase of COVID-19 (ambulant or discharged). **(c)** Cases from the Population-Based Platform (POP). **(d)** Cases from the High-Resolution Platform (HAP).

We investigated the impact of the used anonymization procedure on associations between parameters by computing four regression models for different sizes of datasets before and after anonymization (Fig. 8). The results of the NAPKON PUFs consistently reflected the trends of the associations found in the respective original datasets.

The odd ratios (ORs) deviated for different sizes of datasets. As an example, in the original dataset, the association between inpatient cases from the SUEP aged older than 59 years and dying during the acute phase was estimated with a minimum of OR = 1.72 with 95%-confidence interval (CI) 0.38–7.49 (154 cases) and a maximum of OR = 4.29 with 95%-CI = 2.47–7.91 (908 cases). Comparing the models derived from the NAPKON PUF with those derived from the original dataset, the ORs and CIs are getting closer to the original results when more cases were included. The median absolute deviations of estimated ORs before and after anonymization for datasets of different sizes were for inpatient cases from the SUEP aged older than 59 years and dying during the acute phase (a) 0.2 with minimum (min) 0 and maximum (max) 1.48 difference (Fig. 8(a)), for 49 to 59 years old inpatient cases of the SUEP and HAP that were in a severe phase having any symptom at three month follow up median = 0.03, min = 0.01 and max = 0.13 (Fig. 8(b)), for female cases from the POP having any symptom at three month follow-up median = 0.01, min = 0, and max = 0.58 (Fig. 8(c)), and for cases from the HAP aged older 59 years having intensive care treatment median = 0.52, min = 0.25, max = 0.91 (Fig. 8(d)).

### Reidentification risk

**Fig. 9:**
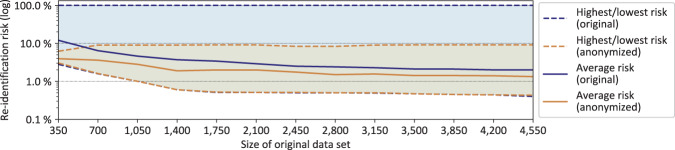
Re-identification risks based on the uniqueness of k-variables before and after anonymization for different sizes of the original dataset (anonymized = PUF).

Figure 9 illustrates how anonymizing the dataset affects the re-identification risk for the patients included. For both, the original and the anonymized dataset, the lowest, highest, and the average re-identification risk is provided. As the original dataset contained unique records, the highest re-identification risk was 100%. In the NAPKON PUF, the highest risk was reduced to not more than 9.09%, as one of the used privacy models required each record to be indistinguishable from at least 10 other records ($1/11=0.\overline{09}$). As expected, the average risk was much lower and further decreased with an increasing number of documented cases. Furthermore, there was no difference in the lowest re-identification risk between the datasets. The original dataset contained records with a low re-identification risk requiring no additional protection.

## Discussion

In this study, we found that statistical bias introduced by anonymization for the NAPKON PUF is small. Descriptive statistics as well as regression analyses showed acceptable differences with only little biases in statistical results. Cases with less frequent characteristics were excluded from the anonymized dataset. However, regression models showed comparable results for the anonymized and the original dataset if parameters with relatively rare values were used as independent variable. The bias decreased as the size of the original dataset increased. Overall, the NAPKON PUF contains only a few variables of the original dataset with a reduced case number, but it preserves important information and a high utility.

We note that statistical results obtained from the original dataset vary with an increasing case number. Small deviations may therefore generally be acceptable. However, since anonymized datasets are likely to contain additional biases and may vary across cohorts with different underlying characteristics, it is important to make the anonymization process transparent when they are shared. In addition to enabling an analysis team to adequately interpret the results for themselves from the anonymized data provided, transparency of the data preprocessing steps is important for research integrity and for making the limitations of studies visible^12^.

Comparing the fraction of cases published in the PUF over time (different sizes of original dataset) with the recruitment rate in NAPKON (https://napkon.de), a relation can be seen between the high recruitment rates in NAPKON in Q4/2020, Q1/2021 as well as Q2/2021 and a larger number of cases that could be included in the PUF in these time periods. This is caused by the fact that the privacy models utilized are based on the principle of ‘hiding in the crowd’^13^. The more cases are included in the original dataset; the more cases can be included in the anonymized dataset as well. Due to a high-granularity data collection in the HAP, the case number was lower than in the SUEP and the POP. This explains why a lower fraction of cases from the HAP can be included in the PUF compared to the other cohorts and why the course of cases included in the PUF flattens out after Q2 2021. Furthermore, the results of the descriptive analyses showed no significant differences in the distribution of gender and quarters of diagnosis in 2020 between the original and the anonymized dataset. For age, disease severity and quarters of diagnosis in 2021 the differences between the two datasets were significant with regard to all of the three cohorts.

The results of this study show that the bias in descriptive characteristics due to anonymization processes can be small. We therefore hypothesize that anonymized data with a comparable complexity may be suitable for various applications. In the following, four possible applications of anonymized data sets are presented, as evidenced by examples from NAPKON. (i) Anonymized data could support researchers in cohort discovery. For the NAPKON PUF, the patient-level information on general characteristics and clinical course severity can help to describe the recruited patient collective, providing insights into the different recruitment strategies in the NAPKON cohorts. (ii) Anonymized datasets further could contribute to facilitate the feasibility process when applying for comprehensive datasets, as the public dataset is an extract from the comprehensive dataset. In NAPKON, researchers can explore the data and feasibility for their research question on their own. A majority of requests for data usage were based mainly on the parameters included in the NAPKON PUF. (iii) Furthermore, anonymized dataset with same parameters originating from different cohorts could contribute to the assessment of the generalizability of results. In NAPKON, the transferability of results from one cohort to another could be assessed by comparing characteristics between the cohorts. For example, we know that the age structure in the SUEP and the HAP differs. Therefore, it can be assumed that the computed OR for age older than 65 years and death in the acute phase of COVID-19 slightly differs in the populations. (iv) In addition, the possibility to explore general patient characteristics in anonymized datasets could enhance the assessment of a present selection bias. For example, patient characteristics of NAPKON could be compared to databases containing data from any reported SARS-CoV-2 infected hospitalized patient to assess the generalizability of results. Although open aggregated descriptive statistics of clinical datasets would already enable the estimation of a selection bias, an open clinical dataset containing patient-level information increases flexibility and empowerment of researchers.

We further showed that an anonymized dataset might reflect trends of associations between parameters. These findings open a new field of application for an anonymized dataset in addition to the use for i - iv. If the research question, as well as the covariates and confounders of the intended analysis are known and well defined, anonymization procedures could be used to create an open dataset specific to pre-defined research questions. Routinely collected clinical data or cohort data could be used to generate an open dataset, overcoming strict privacy regulations and logistical as well as personal costs. In particular, for regression analyses, where the number of variables included is often limited, a small anonymized dataset may be sufficient. We therefore performed simple regression analysis to show the impact of even a small public dataset.

Our results confirm findings from other studies analyzing the impact of anonymizing real-world data for use in real-world contexts, of which, however, there are very few to our knowledge. In the LEOSS project, it was shown that the association between age and death could be replicated in the anonymized dataset with regard to significance and trend of ORs^11^. However, clinical data collected in LEOSS are also comparable to the NAPKON data (less granularity in LEOSS)^14^. This was one reason why we decided to use the principles of the LEOSS anonymization process in this study. In a dataset from the social sciences, a study showed that statistical bias introduced by k-anonymity with k = 5 and six key variables (four patient characteristics, two activity variables) was small^15^.

Our analyses performed on the NAPKON PUF demonstrate that for specific usage scenarios the bias due to anonymization of a reduced dataset may be acceptably small. However, there are limitations regarding the complexity of the dataset and the generalizability of the results to other use cases. (i) The extent to which the chosen anonymization method affects a dataset and subsequent analyses must be examined on a case-by-case basis, which is why our findings cannot necessarily be generalized to other datasets and analyses. In particular, it is difficult to make a statement about the transferability of our results a priori. (ii) In addition, the configuration of the anonymization process must be assessed on case-by-case basis. Publishing more sensitive information, such as information on additional infections with the human immunodeficiency virus, may require more stringent anonymization techniques. (iii) Furthermore, the assessment of the bias introduced by anonymization also needs to be performed from the perspective of individual usage scenarios. In a public clinical dataset used for research on discrimination or underprivileged sub-populations anonymization may mask the severity of disparity^16^. (iv) Moreover, for generating the NAPKON PUF, it was necessary to reduce the datasets’ complexity from the beginning. This resulted in a subset of 15 variables. Some variables, such as age or clinical states defined by the WHO Clinical Progression Scale^17^, underwent a categorization to reduce their granularity. By reducing the datasets’ complexity, the number of cases withhold by the anonymization process can be reduced. However, this limits the possibilities of complex statistical analyses as some variables needed are missing or too much generalized for accurate evaluation.

The NAPKON PUF is another practical example of an anonymized dataset with high utility. Nevertheless, data protection legislation still complicates the publication of individual-level anonymous data in Germany and other countries. Therefore, further progress is needed to establish a more standardized way of data anonymization to provide a solid bridge between legal requirements and technical implementation options. As a next step, a standardized framework could be supported by legal opinions, helping to remove uncertainty of whether datasets can be considered legally anonymous.

In addition to statistical considerations regarding the utility of an anonymized dataset, the economic and social benefits must be emphasized. Anonymized clinical data can be easily shared resulting in maximal benefit. In comparison to that, pseudonymized clinical data are access restricted and protected so that time-consuming and potentially costly use and access processes are necessary. Complex data sets like in NAPKON are usually particularly costly to generate and hence often require public funding. Access to anonymized data can help to justify the cost of data collections^18^ and data access is expedited, which is particularly important in times of pandemics. Furthermore, anonymization could improve the cost-effectiveness in case of scarce scientific resources, as anonymized datasets without access restrictions do not need continuous financial expenditures for data distribution^18^.

Finally, we applied a mathematical anonymization procedure to a large clinical dataset containing demographics and clinical information on COVID-19 disease courses. In our study, the statistical bias introduced by anonymization was small. Therefore, we advocate for the use of anonymized clinical datasets in research, supporting use cases such as feasibility analyses or pre-defined non-complex statistical analyses. However, it is difficult to estimate the impact of anonymization in novel datasets a priori. Therefore, statistical interpretation of an anonymized dataset should be carried out with caution.

## Methods

We investigated the statistical bias due to anonymization within the PUF from the NAPKON cohort. The open dataset contains clinical data from SARS-CoV-2 infected patients treated in hospitals, by general practitioners or infected individuals that were identified and contacted via the local public health authorities, collected in three sub-cohorts.

### Ethical statement

NAPKON was approved by local ethics committees of participating sites (primary approval for the SUEP: Ethics Committee of the Department of Medicine at Goethe University Frankfurt (local ethics ID approval 20–924), for the HAP: Ethics Committee of the Charité – Universitätsmedizin Berlin (local ethics ID approval EA2/226/21 and EA2/066/20), for the POP: Ethics Committee of the Department of Medicine at Christian-Albrechts-University Kiel (local ethics ID approval D 537/20)). Patients consent was obtained for data collection, storage, and processing. The data from the anonymized PUF were considered anonymous. The PUF did not contain directly personal information and the re-identification risk was lowered applying the anonymization pipeline.

### NAPKON cohort platforms

The Cross-Sectoral Platform (SUEP) cohort recruits SARS-CoV-2 infected patients from university and non-university medical centers as well as outpatient settings across 40 study sites, which are followed up over a 12-month period. Both pediatric and adult patients are included in the SUEP. Additional cases from the CORKUM cohort were subsequently added to the SUEP dataset^19^. Part of the cases were already recruited at the beginning of 2020. The High-Resolution Platform (HAP) cohort follows a deep phenotyping protocol at eleven university sites and has established follow-up investigations up to 36 months after initial COVID-19 diagnosis. Cases from the preceding Pa-COVID study^20^ were also added to the HAP dataset, including data from patients recruited since the beginning of 2020. The Population-Based Platform (POP) cohort differs from the other two cohorts in that it contains retrospectively collected data from the acute course and prospectively collected data, imaging data, and biosamples from six to 12 months after the initial COVID-19 diagnosis. It focuses on health consequences of SARS-CoV-2 infection in the general adult population^21^.

### Dataset

The dataset used for the evaluation of the statistical effects of anonymization (NAPKON PUF) was extracted from the comprehensive clinical dataset of NAPKON (original dataset), including all cases documented or integrated from 2020-11-01 to 2022-03-15. 15 variables were included in the NAPKON PUF, containing demographic variables, cohort information, and clinical course and outcome parameters (Table 2). Table 3 compares the different features of the NAPKON PUF and the original dataset.

**Table 2:** Assessment of the re-identification risk associated with individual variables.

VARIABLE	Categories	R	A	D	Is Key
**Age at diagnosis**	< = 17 years, 18–39 years, 40–59 years, 60–79 years, > = 80 years	3	3	3	Yes (9)
**Gender**	Male, female, diverse, unknown/missing	3	3	2	Yes (8)
**Quarter first diagnosis**	Q1, Q2, Q3, Q4, unknown/missing	1	3	2	Yes (6)
**Year first diagnosis**	2020, 2021, 2022, 2023, unknown/missing	1	3	2	Yes (6)
**Cohort**	HAP, POP, SUEP	3	3	2	Yes (8)
**Mild disease phase**	Yes, no, unknown/missing	1	2	1	No (4)
**Moderate disease phase**	Yes, no, unknown/missing	1	2	2	No (5)
**Severe disease phase**	Yes, no, unknown/missing	1	2	2	No (5)
**Patient status at end of acute phase**	Discharged, ambulant, referral/transfer, dead, unknown/missing	1	2	2	No (5)
**Hospitalization during acute phase**	Yes, no, unknown/missing	1	2	2	No (5)
**Intensive care treatment**	Yes, no, unknown/missing	1	2	2	No (5)
**Invasive ventilation**	Yes, no, unknown/missing	1	1	2	No (4)
**Availability of 3- month follow-up**	Yes, no/not yet	1	2	1	No (5)
**Ability to work at 3- month follow-up**	N/a, yes, no, unknown/missing	1	2	2	No (5)
**Any symptom at 3- month follow-up**	N/a, yes, no, unknown/missing	1	1	2	No (4)

Key variables are defined by R (replicability) + A (availability) + D (distinguishability) >5. 1 = low risk, 2 = medium risk, 3 = high risk. HAP = High-Resolution Platform (hospitalized patients only), POP = Population-Based Platform (retrospective documentation after SARS-CoV-2 infection), SUEP = Cross-Sectoral Platform (in- and outpatients).

**Table 3:** Comparison of different features of the NAPKON PUF and its original dataset.

	NAPKON PUF	Original NAPKON dataset
**Number of variables**	15	>2.000
**Cases included**	3,904	4,562
**Highest re-identification risk**	9,09%	100%
**Accessibility**	public	for selected scientists after application
**Linkage with additional data**	No further linkage possible	Linkage to further data, image data and biosamples possible
**Usage**	Feasibility checks, basic statistics, cohort descriptions	Comprehensive in-depth analyses

### Variable selection

The variables of the NAPKON PUF are based on the parameters defined in the German COVID-19 core dataset ‘the German Corona Consensus Dataset (GECCO)’^22^. The demographic variables provide a basic overview of the cohorts. By specifying the cohort of included patients, the case numbers can be queried on a cohort-specific basis. This is particularly important, because the cohorts cover different health sectors and data of varying depth. Therefore, not all cohorts are suitable for answering all research questions. For example, the SUEP captures the acute course in the outpatient setting, whereas the HAP only includes patients from the inpatient setting. Therefore, for questions intended to cover outpatient cases, it is possible to filter specifically by “SUEP” and “no hospitalization”. The WHO Clinical Progression Scale focuses on ventilation parameters. Categorization into mild, moderate, and severe does not allow a precise conclusion to be made about an intensive care stay, since the use of different ventilation modalities is possible in normal or intensive care units, depending on the hospital. In addition, other reasons such as the need for dialysis also lead to an intensive care stay for a COVID patient who is not ventilated. Therefore, intensive care treatment is requested individually in the PUF. In addition, the severe phase of the WHO Clinical Progression Scale includes patients with non-invasive ventilation (NIV), high flow and mechanical ventilation. To delineate the number of patients receiving invasive ventilation, this variable was additionally added to the PUF.

In addition to the acute phase of the COVID-19 disease, the long-term outcome plays a main role in scientific analyses, as the long-term consequences of the disease are not yet clear^23,24^. Furthermore, clinical courses differ with regard to SARS-CoV-2 variants^25,26^. The quarter of first COVID-19 diagnosis can help with the assignment to a certain variant. Therefore, asking for the ability to work and the persistence of symptoms three months after original infection greatly enhances the informative strength of the anonymous dataset. The availability of the 3-months follow-up is captured in the dataset (retrospective documentation for POP) and it is particularly important for feasibility queries.

### Anonymization

The anonymization pipeline used for the NAPKON PUF is based on the qualitative and quantitative principles developed for the public dataset of LEOSS^11^. LEOSS is one of the largest COVID-19 registries in Germany with comprehensive clinical data of mainly hospitalized SARS-CoV-2 infected patients^14,27^.

Analogously to the approach implemented for LEOSS, we used the method by Malin *et al*.^28^ and rated all variables along the axes replicability, availability and distinguishability (1 = low risk, 2 = medium risk, 3 = high risk). In the following the rating for two variables is explained exemplarily. The age categorization hardly changes over the observational period (replicability = high risk). The age is often known and can be determined by appearance (availability = high risk), and there is a great variation within the society (distinguishability = high risk). The need of intensive care treatment, from a medical point of view, is only slightly likely to occur repetitively, as the forms of treatment change in the course of time (replicability = low risk). However, for long hospital stays with known absence and possible subsequent rehabilitation, the need for intensive treatment could be suspected (availability = medium risk). Intensive care treatment as a binary variable has low distinguishability, but median discriminability is assessed as only about one-fifth of cases are documented with intensive care (distinguishability = medium risk).

Based on the rating we assessed which variables should be considered “key variables” and need to be modified to protect records from singling out and linkability. Table 2 shows the result of the assessment and the key variables (variables with a score >5) identified. All remaining variables (i.e. non-key variables) are considered sensitive information and will be transformed to protect against inference.

To protect records from singling out, linkability, and inference, the anonymization pipeline used for the NAPKON PUF requires records to satisfy the following criteria to be released: (i) k-anonymity with k = 11 for all key variables (i.e. “age at diagnosis”, “gender”, “quarter first diagnosis”, “year first diagnosis”, “cohort”) and (ii) t-closeness with t = 0.5 for sensitive variables (i.e. “mild disease phase”, “moderate disease phase”, “severe disease phase”, “patient status at end of acute phase”, “intensive care treatment”, “invasive ventilation”, “ability to work at 3-month follow-up” and “any symptom at 3-month follow-up”). The variables “availability of 3-month follow-up” and “hospitalization during acute phase” are not explicitly protected by t-closeness, as they are perfectly correlated with other sensitive variables and thus implicitly protected as well. Further, the anonymization pipeline must guarantee that the criteria mentioned above also hold for continuous releases of new data.

### Evaluation of statistical properties

We evaluated the impact of the implemented anonymization procedures by comparing descriptive statistics and associations in the dataset before and after anonymization. We computed a regression model for each cohort and, in addition, a regression model that combined data from the SUEP and the HAP to analyze the effects of anonymization for the NAPKON cohort in general. The medical validity of analyses using all cohort data would have been limited due to different cohort populations and recruitment strategies. Additionally, we assessed the impact of anonymization for datasets of different sizes, ranking cases by the date of their diagnosis. In doing so, we intended to simulate the scenario of a continuous data release starting with few cases. The month of recruitment, when cases were included in the NAPKON cohort, can be found on the NAPKON homepage (https://napkon.de).

Descriptive statistics were computed using relative frequencies, and distribution of variables before and after anonymization were compared using Chi-Squared test, defining P < 0.05 as statistically significant. The associations were computed using logistic regression models, adjusting for age and gender. The odds ratios and confidence intervals were presented. Cases with unknown/missing data were excluded. To verify the effect of the implemented anonymization methods of the re-identification risk, we computed highest, average, and lowest re-identification risk for the dataset before and after anonymization, again following the approach developed for LEOSS^11^. The risk was calculated based on the sizes of groups of records which are indistinguishable from one another in regard to the attributes which we assume could be used to identify an individual, i.e. key variables.

## Data Availability

A current version of the NAPKON public dataset is released as a CSV file on the NAPKON website (https://napkon.de/statistik/). In addition to the dataset, the homepage offers to explore the dataset in more detail by presenting regularly updated figures of descriptive statistics. The NAPKON dataset used for these analyses is published on Zenodo^29^. The original NAPKON dataset is available from the NAPKON Use and Access Commitee but restrictions apply to the availability of these data, which were used under license for the current study, and so are not publicly available.

## Code availability

The code developed for the NAPKON public dataset is available as open-source software^30^.
